# Zafirlukast is a broad‐spectrum thiol isomerase inhibitor that inhibits thrombosis without altering bleeding times

**DOI:** 10.1111/bph.15291

**Published:** 2021-01-04

**Authors:** Lisa‐Marie Holbrook, Shirley J. Keeton, Parvathy Sasikumar, Sophie Nock, Justine Gelzinis, Elizabeth Brunt, Sarah Ryan, Megan M. Pantos, Christina A. Verbetsky, Jonathan M. Gibbins, Daniel R. Kennedy

**Affiliations:** ^1^ Institute for Cardiovascular and Metabolic Research, School of Biological Sciences University of Reading Reading UK; ^2^ School of Cardiovascular Medicine and Sciences King's College London London UK; ^3^ Centre for Haematology Imperial College London London UK; ^4^ College of Pharmacy and Health Sciences Western New England University Springfield Massachusetts USA

**Keywords:** platelets, protein disulphide isomerases, redox, thrombosis, zafirlukast

## Abstract

**Background and Purpose:**

Multiple members of the thiol isomerase (TI) family of enzymes are present in and released by platelets. Inhibition of these enzymes results in diminished platelet responses, aggregation, adhesion and thrombus formation. Recently, the therapeutic potential of TI inhibition has been recognised and drug‐development technologies were used to identify selective small molecule inhibitors. To date, few pan‐TI inhibitors have been characterised and the most studied, bacitracin, is known to be nephrotoxic, which prohibits its systemic therapeutic usage.

**Experimental Approach:**

We therefore sought to identify novel broad‐spectrum inhibitors of these enzymes and test their effects in vivo. A total of 3,641 compounds were screened for inhibitory effects on the redox activity of ERp5, protein disulphide isomerase (PDI), ERp57, ERp72 and thioredoxin in an insulin turbidity assay. Of the lead compounds identified, zafirlukast was selected for further investigation.

**Key Results:**

When applied to platelets, zafirlukast diminished platelet responses in vitro. Zafirlukast was antithrombotic in murine models of thrombosis but did not impair responses in a model of haemostasis. Since TIs are known to modulate adhesion receptor function, we explored the effects of zafirlukast on cell migration. This was inhibited independently of cysteinyl LT receptor expression and was associated with modulation of cell‐surface free thiol levels consistent with alterations in redox activity on the cell surface.

**Conclusion and Implications:**

We identify zafirlukast to be a novel, potent, broad‐spectrum TI inhibitor, with wide‐ranging effects on platelet function, thrombosis and integrin‐mediated cell migration. Zafirlukast is antithrombotic but does not cause bleeding.

**Abbreviations**
CysLT: cysteinyl leukotriene
CysLTR: cysteinyl leukotriene receptor
MPB: *N* ^α^‐(3‐maleimidylpropionyl)biocytin
PDI: protein disulphide isomerase
TI: thiol isomerase
ZFL: zafirlukast

**What is already known**

- Platelets contain multiple TIs which are key to the regulation of platelet activation and thrombosis.
- No pan‐TI inhibitors that are safe and tolerated in vivo are therapeutically available.

**What this study adds**

- Zafirlukast inhibited multiple TI and platelet function in vitro and in vivo.
- Zafirlukast inhibits cell migration through redox modifications of cell‐surface proteins.

**What is the clinical significance**

- Zafirlukast shows antithrombotic effects without affecting haemostasis.

## 1 INTRODUCTION

Thrombosis‐related pathologies such as myocardial infarction, stroke, pulmonary embolism or deep vein thrombosis are the primary cause of mortality for much of the Western world (Furie & Furie, 2012), highlighting the importance of identifying new therapeutic targets to treat these diseases. One such interesting target is protein disulphide isomerase (PDI), which, when inhibited, attenuates both arterial and venous thrombosis, unlike current clinically used therapies (Cho et al., 2012; Jasuja et al., 2012). Protein disulphide isomerase is the most well‐studied member of a family of thiol isomerases (TIs) that includes ERp5 (Jordan et al., 2005), ERp57 (Holbrook et al., 2012), ERp72 (Holbrook et al., 2018), ERp44, TMX3 and thioredoxin (Holbrook et al., 2010), which are found on the surface of platelets and involved in the propagation of platelet activation and subsequent thrombus formation. Using inhibitory antibodies that are selective for cell‐surface protein disulphide isomerase (Essex & Li, 1999; Lahav et al., 2002), ERp5 (Jordan et al., 2005), ERp57 (Holbrook et al., 2012) or ERp72 (Holbrook et al., 2018), thiol isomerase (TI) inhibition has been shown to result in decreased platelet aggregation, granule secretion, integrin activation, integrin‐mediated adhesion and thrombus formation.

Much work has focused on identifying specific small molecule inhibitors of protein disulphide isomerase, including quercetin analogues (isoquercetin and rutin) (Jasuja et al., 2012; Stopa et al., 2017), BAP2 analogues (Yang et al., 2019) and bepristats (Bekendam et al., 2016; Khodier et al., 2013), which all inhibit thrombus formation and fibrin generation in mice, while isoquercetin has been demonstrated to have clinical potential in phase II clinical studies (Stopa et al., 2017; Zwicker et al., 2019).

We have previously shown that while inhibition of specific platelet thiol isomerase results in broadly similar impact on platelet activation and thrombosis, these effects are not identical suggesting different substrates and/or mechanisms of action and therefore each thiol isomerase could be an independent target (Holbrook et al., 2012; Holbrook et al., 2018). Importantly, the effects of platelet thiol isomerases are non‐redundant, where addition of one family member is unable to rescue the absence of another (J. Zhou et al., 2017), again suggesting distinctive modes of action. Given the non‐redundant roles of these enzymes, it is important to determine if broad‐spectrum inhibition is an effective therapeutic strategy and to determine its effects in vivo with respect to prevention of thrombosis without compromising haemostasis. To date, the only known broad‐spectrum thiol isomerase inhibitor is the antibiotic bacitracin, which is limited to topical use due to nephrotoxicity (Michie, Zintel, Ma, Ravdin, & Ragni, 1949). Thus, in this study, we sought to identify new family‐wide (pan) inhibitors that may show promise for human disease prophylaxis. We report that the structurally related cysteinyl LT (CysLT ) receptor antagonists zafirlukast and montelukast are powerful inhibitors of thiol isomerase activity. Zafirlukast, the more potent inhibitor, was found to modulate platelet function and integrin function in vitro and inhibit thrombosis in mice while bleeding was unaffected.

## 2 METHODS

Cross‐linked collagen‐related peptide (CRP‐XL) was purchased from Prof Richard Farndale (University of Cambridge, Cambridge, UK) and Horm collagen (type I) from Nycomed, Germany. Chronolume ATP standard reagents were from Chronolog (Havertown, PA, USA). Montelukast (MTL), zafirlukast (ZFL), bovine insulin and bacitracin were purchased from Sigma Aldrich (Poole, UK). Fura‐2 AM, normal goat serum, *N* ^α^‐(3‐maleimidylpropionyl)biocytin (MPB) and phalloidin Alexa‐Fluor 568 conjugate were from Thermo Fisher Scientific (Leicestershire, UK). Anti‐GPIb DyLight 649‐conjugated antibody (Cat# X649) and anti‐mouse P‐selectin‐FITC conjugate (Cat# D200) were from Emfret Analytics (Germany). Anti‐human fibrinogen‐FITC conjugate was from Dako (Cambridgeshire, UK) and anti‐human P‐selectin‐PE conjugate (Cat# 555524, RRID:AB_395910) was obtained from BD Biosciences (Oxford, UK). Streptavidin‐HRP conjugate (Cat# ab7239, RRID:AB_305786) was from Abcam (Cambridge, UK). Vectashield antifade mounting medium was from Vector Laboratories (Cat# H‐1000, RRID:AB_2336789, Peterborough, UK).

### 2.1 Insulin‐based turbidometric assay

Initial compound library screening was performed in an insulin turbidity assay adapted for high‐throughput screening (Holmgren, 1979; Smith et al., 2004). The reduction of the disulphide bond in insulin by a thiol isomerase results in the separation of the A and B chains and precipitation, which is measured by spectrophotometry at a wavelength of 650 nm. Using Corning 3891 well plates (with a final volume of 10 μl), a final concentration of 10 μg·ml^−1^ of ERp57, 125‐μM insulin and 2‐mM EDTA in potassium phosphate buffer (100 mM) was added to each experimental well. Bacitracin (3 mM) was added to select wells as a positive control and vehicle plates served as a negative control. Experimental compounds were pin transferred to the wells at a final concentration of 30 μM and plates shaken. After incubation for 10 min, 0.3‐mM DTT was added to propagate enzyme activity. Following reaction for 1 hr at 37°C, plates were read using a PerkinElmer Envision plate reader (PerkinElmer, Waltham, MA). All compounds were tested in duplicate at the ICCB‐Longwood Screening Facility at Harvard Medical School.

Lead compounds were diluted in a 6‐point dose curve (concentrations depending on the observed effects in the initial screen) and insulin turbidity was measured kinetically each minute for 75 min using a Spectramax M3 microplate reader (Molecular Devices, Sunnyvale, CA). Specificity assay also utilised the insulin turbidity assay to determine specificity of inhibition; ERp57 was replaced with PDI (10 μg·ml^−1^), ERp5 (30 μg·ml^−1^), ERp72 (10 μg·ml^−1^) or thioredoxin (10 μg·ml^−1^), to determine the ability of lead compounds to inhibit these enzymes.

### 2.2 Platelet preparation and stimulation

Washed human platelets from consenting, drug‐free donors were prepared by differential centrifugation as described previously (Holbrook et al., 2018) and suspended to a density of 4 × 10^8^ cells·ml^−1^ in Tyrodes‐HEPES buffer (134‐mM NaCl, 2.9‐mM KCl, 0.34‐mM Na_2_HPO_4_, 12‐mM NaHCO_3_, 20‐mM HEPES, 1‐mM MgCl_2_ and 5‐mM glucose, pH 7.3). Prior to stimulation, platelets were incubated with zafirlukast or vehicle (DMSO, 0.1% v/v) for 5 min. Platelets were stimulated with agonists for 180 s in a lumi‐aggregometer (Chronolog, Havertown, PA, USA) with continuous stirring. Mouse blood was obtained on the day of experimentation by cardiac puncture following termination by increasing concentrations of CO_2_. PRP was isolated from anticoagulated (4% w/v sodium citrate) blood by centrifugation at 200 *g* for 8 min. Platelets were pelleted by centrifugation at 1,000 *g* for 5 min in the presence of 100 ng·ml^−1^ PGI_2_ and re‐suspended to a density of 2 × 10^8^ cells·ml^−1^ in Tyrodes‐HEPES buffer.

### 2.3 Platelet granule secretion and calcium mobilisation measurements

P‐selectin exposure in human and mouse platelets was measured by flow cytometry and calcium mobilisation in human platelets assayed as described previously using Fura‐2 AM (Holbrook et al., 2018). ATP secretion from dense granules was measured using lumi‐aggregometry (Holbrook et al., 2012).

### 2.4 Assessment of arterial thrombus formation and tail bleeding assay

Male C57BL/6J mice (RRID:IMSR_JAX:000664 bred in house, at age 4–5 weeks with a weight range of 19–25 g) were anaesthetised by intraperitoneal injection of ketamine (125 mg·kg^−1^), xylazine (12.5 mg·kg^−1^) and atropine (0.25 mg·kg^−1^). Platelets were labelled by intravenous infusion of DyLight 649‐conjugated anti‐GPIb platelet labelling antibody (0.2 μg·g^−1^ body weight). Following exposure of the testicular cremaster muscle, vehicle (DMSO, 0.1% v/v) or zafirlukast (20 μM·ml^−1^ of blood volume) was infused intravenously and following a 5‐min incubation period, arteriole wall injury was induced by ablation laser (Micropoint, Andor Technology, Belfast, UK). Thrombi were observed using an Olympus BX microscope (Olympus, Essex, UK) and a Hamamatsu (Hamamatsu Photonics, Hertfordshire, UK) CCD camera and data analysed using Slidebook Software version 5.0 (Intelligent Imaging Innovations, Denver, USA) (Falati et al., 2006; Holbrook et al., 2018; Holbrook et al., 2012). Mice were killed using Schedule 1 approved methods at the end of the experiment. For tail bleeding assays, female C57BL/6J mice, bred in house (RRID:IMSR_JAX:000664, 6–8 weeks old, 21–30 g), were anaesthetised by intraperitoneal injection of ketamine (125 mg·kg^−1^), xylazine (12.5 mg·kg^−1^) and atropine (0.25 mg·kg^−1^), and drug or vehicle infused into the femoral vein 5 min prior to tail biopsy. Following drug treatment, 0.5 cm of tail tip was excised and blood collected into sterile PBS. Time to bleeding cessation was recorded. Mice were killed when bleeding had ceased or at 20 min' post tail tip excision. All animal experiments were blinded for both the experimental treatment and the analysis. Animal experiments were approved by the University of Reading Local Ethical Review Panel and authorised by the UK Home Office. Animal studies are reported in compliance with the ARRIVE guidelines (Percie du Sert et al., 2020) and with the recommendations made by the *British Journal of Pharmacology* (Lilley et al., 2020).

### 2.5 Cell migration scratch assays

MDA‐MB‐231 (ATCC Cat# HTB‐26, RRID:CVCL_0062) or HEK293T (ATCC Cat# CRL‐3216, RRID:CVCL_0063) were seeded into wells of a 12‐well plate (fibronectin coated, 10 μg·ml^−1^) and grown to confluence in DMEM supplemented with 10% (v/v) FBS and 1% (v/v) penicillin–streptomycin. Following aspiration of media, a vertical scratch was performed to bisect the well and detached cells were removed by PBS washes. Vehicle (DMSO, 0.1% v/v) or zafirlukast was added to each well in the presence of DMEM with 1% (v/v) FBS, and wells were imaged at 1, 4, 16, and 37 hr (MDA‐MB‐231) or 1, 4, 20 or 40 hr (HEK293T) post‐scratch using a Zeiss A1 Axiovert Inverted Epifluorescent microscope with a ×5 Planar Plan Neofl Ph1 0,15 ∞ /0,17 objective. Images were analysed using Zeiss Axiovision software, and closure areas calculated using Fiji ImageJ. At 37/40 hr post‐scratch, cells were fixed in 4% (v/v) paraformaldehyde for 20 min and permeabilised with 0.2% (v/v) Triton X‐100 for 10 min. Cells were stained with phalloidin‐568 (dilution of 1:250, in PBS) for 1 hr and following washes, counterstained with DAPI (1:1,000) for 20 min. Cells were washed in PBS and mounted using Vectashield H‐1000.

### 2.6 Labelling of cell‐surface thiols

MDA‐MB‐231 or HEK293T cells were cultured and treated as described before. Four hours post‐treatment, cells were washed with PBS, and free thiols labelled by incubation with MPB (100 μM) for 20 min. Free label was removed by multiple PBS washes, and cells were lysed in reducing sample treatment buffer for SDS‐PAGE analysis. Thiol labelling was detected using streptavidin‐HRP conjugate (1:10,000 dilution in 5% [w/v] BSA/TBS‐T), and chemiluminescence captured following the addition of ECL substrate.

### 2.7 Proliferation assays

MDA‐MB‐231 or HEK293T cells were seeded at a density of 2.5 × 10^5^ cells per well in a six‐well cell culture plate and grown for 24 hr. Vehicle (DMSO, 0.1% v/v) or zafirlukast was then added for a further 72 hr. Following drug treatment, wells were washed and cells manually counted by haemocytometer or automatically using a Coulter counter (Beckman Coulter, CA, USA). Cell growth inhibition was calculated by comparing the counts of drug treated cells with untreated (control) cells as described previously (Kennedy et al., 2007).

### 2.8 Data analysis

Data were analysed using GraphPad Prism software, and statistical analysis was performed using one‐way ANOVA with a Dunnett’s post‐test or Student's unparied *t*‐test as appropriate. Intravital data were additionally analysed using Mann–Whitney *U* test. Power calculations were used for determining the appropriate sample size for tail bleeding assays with the following criteria used: a calculated effect size of 1.78, significance level of 0.05, and a power of 95%. Non‐normalised data were used for statistical analysis. Data where *P* < 0.05 were considered significant. Post hoc tests were only conducted if F in ANOVA analyses achieved P < 0.05 and there was no significant variance in homogeneity. Sample sizes subjected to statistical analysis were at least 5 per group (n=5), where n=number of independent values. The data and statistical analysis comply with the recommendations of the *British Journal of Pharmacology* on experimental design and analysis in pharmacology.

### 2.9 Nomenclature of targets and ligands

Key protein targets and ligands in this article are hyperlinked to corresponding entries in the IUPHAR/BPS Guide to PHARMACOLOGY http://www.guidetopharmacology.org, and are permanently archived in the Concise Guide to PHARMACOLOGY 2019/20 (Alexander et al., 2019).

## 3 RESULTS

### 3.1 Zafirlukast is a potent, broad‐spectrum thiol isomerase inhibitor

**FIGURE 1:**
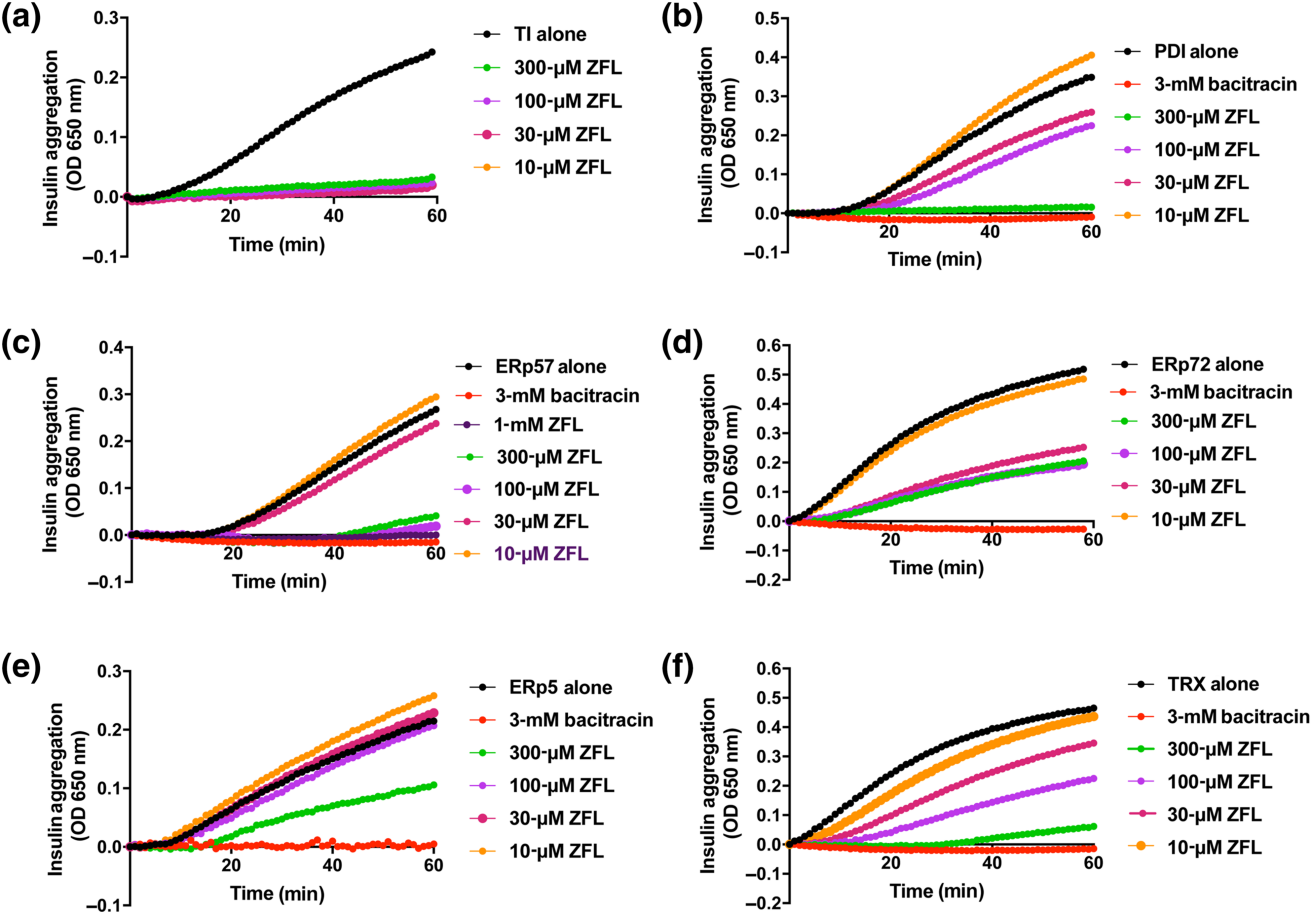
Zafirlukast (ZFL) is a broad‐spectrum thiol isomerase inhibitor. A total of 3,641 compounds were screened for inhibitory effects on the redox activity of ERp5, protein disulphide isomerase (PDI), ERp57, ERp72 and thioredoxin (TRX), all assayed at 10 μg·ml^−1^ except ERp5 which was assayed at 30 μg·ml^−1^. ZFL was selected for further investigation. The ability of zafirlukast to inhibit thiol isomerase activity and its specificity was measured by insulin turbidity assay in which insulin chain precipitation through disulphide bond reduction was measured at a wavelength of 650 nm in a spectrophotometer at 37°C. (a) Enzyme assays performed in the absence of enzyme with drug only. Zafirlukast concentration dependently inhibits (b) PDI, (c) ERp57, (d) ERp72, (e) ERp5 and (f) thioredoxin (TRX) activity

A total of 3,641 compounds were assayed for their ability to inhibit ERp57 activity by insulin turbidity assay. Initial screen data were cross‐checked against databases of compounds with known false positive readouts, and consequently, 11 compounds selected as potential leads. These compounds were assayed for time‐dependent enzyme‐inhibitory activity of the other known platelet thiol isomerases PDI, ERp5, ERp72 and thioredoxin. A compound of particular interest, zafirlukast, from the CysLT receptor antagonist family was found to exert inhibition on all enzymes tested (Figure 1). Additionally, the effects of the related CysLT receptor antagonist montelukast were explored (Figure S1). The insulin turbidity assay was also repeated to measure the effects of zafirlukast in this assay in the absence of enzyme. Zafirlukast did not cleave insulin or alter its optical properties in solution (Figure 1a).

Despite zafirlukast (Figure 1b–f) and montelukast (Figure S1) exhibiting differing potencies, both compounds (at the highest concentration tested of 300 μM) were able to almost entirely ablate the enzyme activity of PDI, ERp57 and thioredoxin.

Both zafirlukast and montelukast are FDA‐approved treatments for the prevention of asthma (marketed as Accolate and Singulair, respectively). Both drugs are clinically tolerated well, can be administered orally for long‐term therapy and show little toxicity. In addition to their role in CysLT receptor function, we demonstrate a novel role for zafirlukast and montelukast as potent thiol isomerase inhibitors. Since zafirlukast showed higher potency than montelukast in the insulin turbidity assay, we focused our functional studies on the use of zafirlukast.

### 3.2 Zafirlukast inhibits platelet aggregation and granule secretion

**FIGURE 2:**
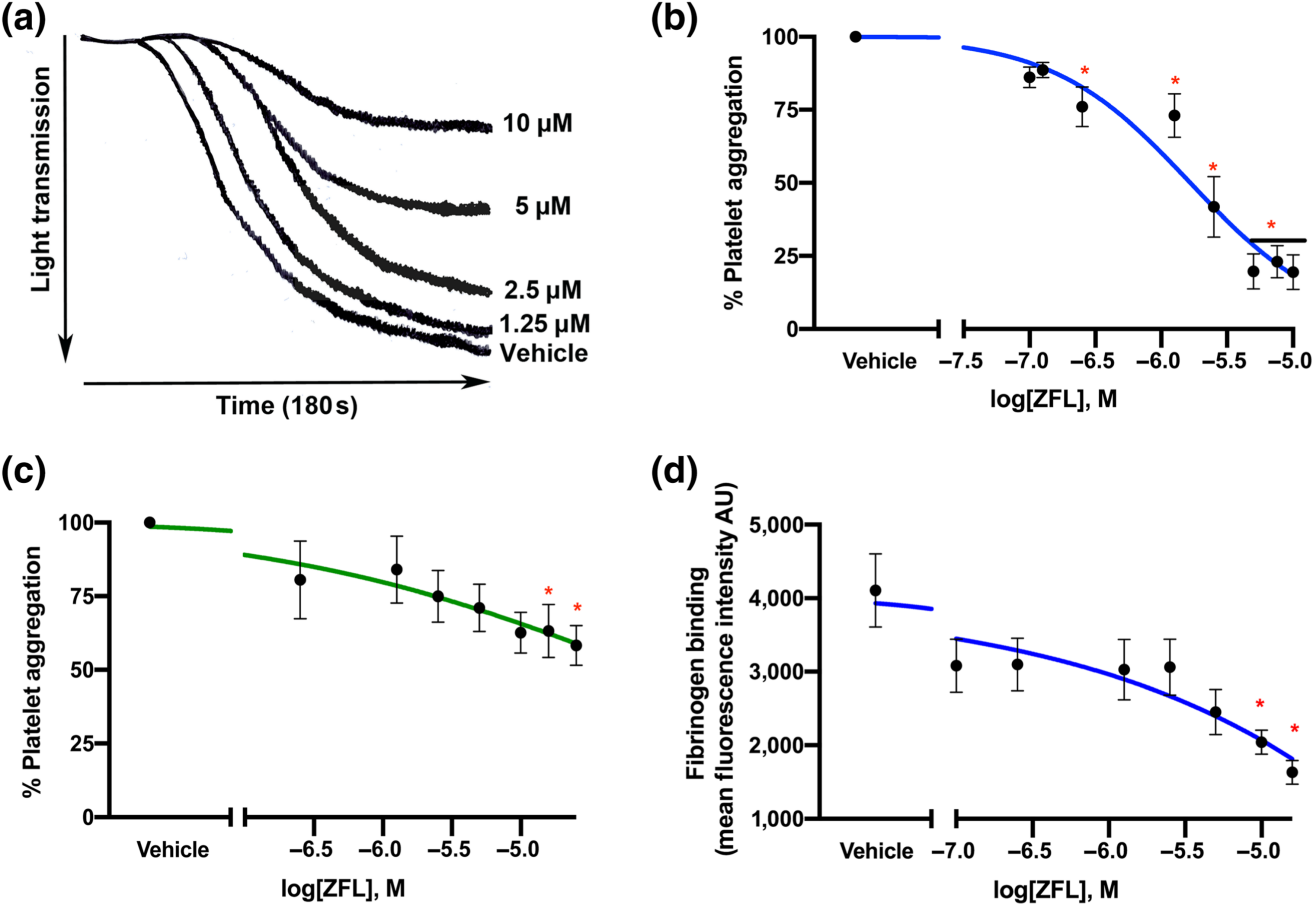
Zafirlukast (ZFL) inhibits platelet aggregation and fibrinogen binding. Washed human platelets (4 × 10^8^ cells·ml^−1^) were incubated with ZFL (0.1–10 μM) or vehicle for 5 min prior to stimulation with (a) collagen (1 μg·ml^−1^) for 180 s in an optical aggregometer. (b) Data were normalised to vehicle, and % inhibition of aggregation (mean ± SEM) was calculated, *n* = 12. (c) Log aggregation response to thrombin (0.1 U·ml^−1^), *n* = 6. (d) Fibrinogen binding was measured by flow cytometry. Platelets (2 × 10^8^ cells·ml^−1^) were incubated with vehicle or zafirlukast (0.1–20 μM) for 5 min prior to stimulation with 0.25 μg·ml^−1^CRP‐XL. Anti‐human fibrinogen‐FITC conjugate was added for 20 min (1:500 dilution), and samples were fixed with 0.2% (v/v) paraformaldehyde; data for 10,000 events were recorded, *n* = 6. Graphs represent mean ± SEM. Data were analysed by one‐way ANOVA, *P < 0.05,

Supplementation of platelet suspensions with the recombinant enzymes PDI (J. Zhou et al., 2015), ERp72 (J. Zhou et al., 2017) or ERp57 (Wu et al., 2012) potentiates platelet aggregation and inhibition or ablation of these enzymes impairs platelet responses (Essex & Li, 1999; Holbrook et al., 2018; Holbrook et al., 2012). The data presented in Figure 1 demonstrate that ZFL acts as a potent inhibitor of multiple thiol isomerases, therefore we sought to confirm the effects of zafirlukast on platelet activation. Platelet aggregation was measured in washed platelet suspensions in the presence of either vehicle (DMSO, 0.1% v/v) or zafirlukast (0.1–10 μM) and stimulated for 180 s with collagen (1 μg·ml^−1^, Figure 2a,b). zafirlukast treatment (10 μM) decreased platelet aggregatory responses by 85% (±4.17%), 5 μM reduced aggregation by 78% (±7.37%), 2.5 μM by 53% (±10.56%), 1.25 μM by 35% (±10.9%) and 0.6 μM decreased aggregation to 35% (±10.66%). Platelet aggregation in response to collagen had an IC_50_ value of 1.66 μM. Platelet aggregation was also measured using thrombin (0.1 U) as an agonist, which showed zafirlukast to be less potent at inhibiting this pathway of platelet activation compared with collagen (calculated IC_50_ of 79.2 μM) (Figure 2c). Raw data for mean platelet responses are presented in Figure S2.

Thiol isomerase inhibition is known to impair integrin α_IIb_β_3_ activation and conformational changes leading to diminished fibrinogen ligation (Holbrook et al., 2018; Holbrook et al., 2012). The effects of zafirlukast on platelet surface fibrinogen binding were therefore measured using flow cytometry with platelet populations gated on unstained and unstimulated cells (mean fluorescence intensity of 178 ± 3.57 AU). Vehicle samples had a mean fluorescence intensity of 4,106 ± 499.2. Zafirlukast pretreatment (10 μM) significantly reduced fibrinogen binding to platelets and therefore integrin activation by 50% (2043 ± 165.4 AU, Figure 2d). A higher concentration of 20‐μM zafirlukast inhibited fibrinogen binding further causing 61% inhibition (1,633 ± 161.8 AU). Incubation with lower concentrations of zafirlukast (0.1–5 μM) showed a trend towards inhibition but did not reach significance.

**FIGURE 3:**
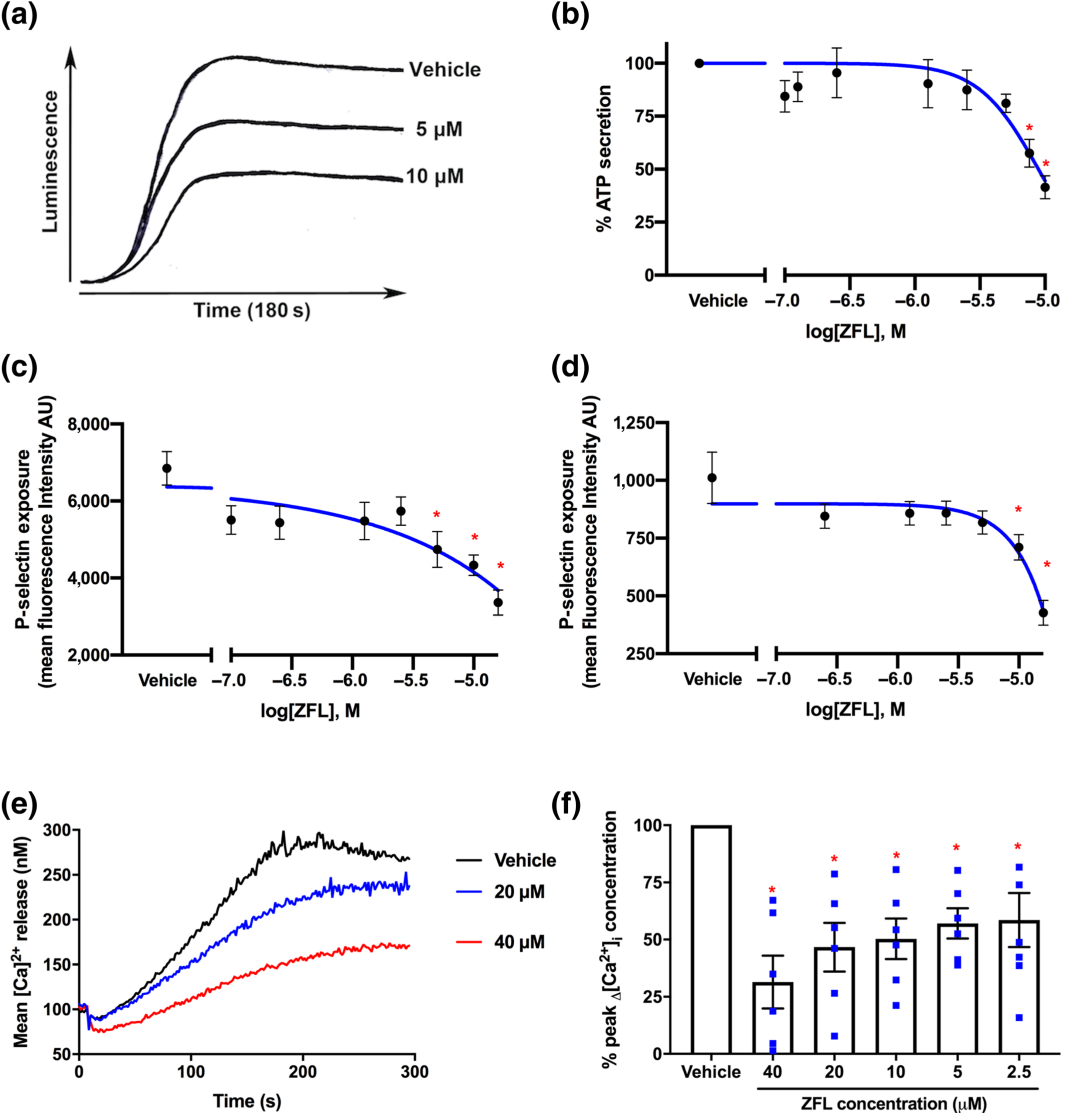
Platelet granule secretion and calcium flux in human and mouse platelets is diminished by zafirlukast (ZFL). Washed human platelets (4 × 10^8^ cells·ml^−1^) were incubated with ZFL (0.1–10 μM) or vehicle for 5 min prior to stimulation with collagen (1 μg·ml^−1^). Chronolume reagent was added for 2 min prior to stimulation, and luminescence recorded. (a) Representative traces of ATP secretion, and (b) is log dense granule secretion values, *n* = 5. (c) For α‐granule secretion, human platelet P‐selectin exposure was measured by flow cytometry. Platelets (2 × 10^8^ cells·ml^−1^) were incubated with vehicle or zafirlukast (0.1–20 μM) for 5 min prior to stimulation with 0.25 μg·ml^−1^ CRP‐XL. Anti‐human P‐selectin PE conjugate was added for 20 min (1:500 dilution), and samples were fixed with 0.2% (v/v) paraformaldehyde, *n* = 6. (d) Following incubation with vehicle or zafirlukast (0.6–20 μM), mouse platelets were stained with anti‐mouse P‐selectin FITC conjugate and stimulated with thrombin (0.1 U·ml^−1^), *n* = 12. For Ca^2+^ mobilisation assays, platelets (4 × 10^8^ cells·ml^−1^) were loaded with Fura‐2 AM and incubated with either vehicle or zafirlukast (2.5–40 μM) for 5 min before addition of CRP‐XL (0.5 μg·ml^−1^), (e) mean Ca^2+^ trace. (f) peak Ca^2+^ concentration normalised to vehicle values, *n* = 6. Graphs represent mean ± SEM; data were analysed by one‐way ANOVA, *P < 0.05

Dense granules secrete secondary mediators of platelet activation such as ADP which underpin platelet activation and aggregation. Previous studies have shown that this process is reliant on thiol isomerase family members (Holbrook et al., 2018; Holbrook et al., 2012; Jordan et al., 2005; Robinson, O'Neill, Kiernan, O'Donoghue, & Moran, 2006). Dense granule secretion was measured in human platelets pre‐incubated with varying concentrations of zafirlukast (0.6–10 μM) and stimulated with collagen (1 μg·ml^−1^). Zafirlukast treatment (10 μM) reduced ATP secretion by 63% (±3.72%) (Figure 3a,b). IC_50_ was calculated to be 8.99 μM for dense granule secretion.

In contrast to the sensitivity of dense granule secretion to thiol isomerase inhibition, α‐granule secretion can be differentially regulated by different members of the thiol isomerase family. ERp5 and ERp72 inhibition results in substantial α‐granule secretion impairment whereas inhibition of ERp57 and PDI has little or no effect, supporting the notion that different thiol isomerases act in subtly different manners (Holbrook et al., 2018; Holbrook et al., 2012; Jordan et al., 2005; Kim et al., 2013). Since platelet aggregation and the requisite integrin activation and fibrinogen ligation are dampened by zafirlukast, it was important to determine if secondary mediator secretion from α‐granules was affected in human (Figure 3c) and mouse platelets (Figure 3d). Unstained and unstimulated platelets were used to gate the platelet population with mean fluorescence intensity values of 305 ± 2.11 (human platelets) and 165.5 ± 1.88 (mouse platelets). P‐selectin exposure in response to cross‐linked collagen‐related peptide (CRP‐XL; 0.25 μg·ml^−1^, human platelets) or thrombin (0.1 U·ml^−1^, mouse platelets) stimulation was unaffected by vehicle treatment (mean fluorescence of 6,845 ± 436.1 AU). Zafirlukast at the highest concentration tested (20 μM) reduced human platelet P‐selectin exposure by 50% (mean fluorescence of 3,364 ± 326.3). Concentration‐dependent inhibition was observed at all concentrations above 5 μM. Mouse platelet α‐granule secretion was also impaired by zafirlukast treatment (20 μM). Mean vehicle fluorescence levels post‐stimulation were 1,012 (±110.9 AU); these were reduced by 57% (427.1 ± 53 AU) by zafirlukast treatment.

Similar to α‐granule secretion, Ca^2+^ mobilisation from intracellular stores is differentially regulated by individual thiol isomerases. Neither ERp5 nor PDI ablation reduces GPVI‐stimulated Ca^2+^ mobilisation, whereas ERp57 and ERp72 inhibition results in delayed responses and lower peak levels of Ca^2+^ (Holbrook et al., 2018; Holbrook et al., 2012; Jordan et al., 2005; Kim et al., 2013). In human platelets, vehicle treatment did not impair calcium mobilisation, whereas, at the highest concentration tested (40 μM), zafirlukast reduced Ca^2+^ mobilisation by 58% (±10.9%) (Figure 3e,f).

### 3.3 Zafirlukast inhibits thrombosis but does not cause bleeding

**FIGURE 4:**
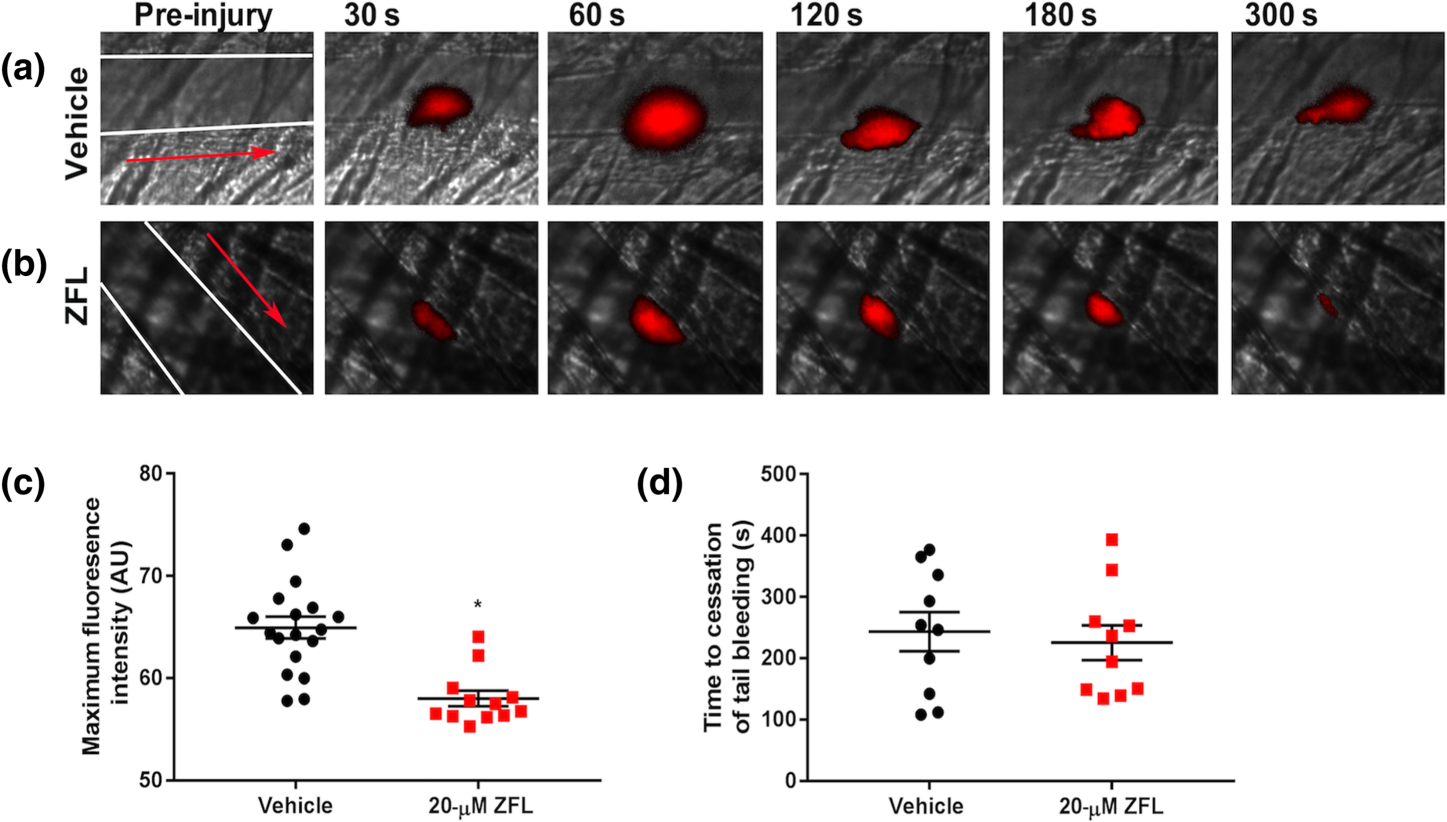
Thrombus formation in vivo is inhibited by zafirlukast with no impact on bleeding times. The effects of zafirlukast (ZFL) on thrombus formation in mice were determined following laser injury of cremaster muscle arterioles and observed by intravital microscopy. Male C57/BL6 mouse platelets were labelled with DyLight 649‐conjugated anti‐GPIb antibody (0.2 μg·g^−1^ body weight) and either vehicle (a) or zafirlukast infused (at a volume required to achieve a circulating concentration of 20 μM) (b). Following laser injury, images were recorded for 5 min. The vessel wall is outlined in each pre‐injury panel, and the red arrow indicates direction of blood flow. (c) Maximum fluorescence intensity of each thrombus formed in vehicle‐treated mice (*n* = 18 thrombi, black circles) or zafirlukast‐treated mice (*n* = 12 thrombi, red squares). (d) The effects of zafirlukast on bleeding were determined by tail bleeding assay. Vehicle or zafirlukast (at a volume required to achieve a circulating concentration of 20 μM) was infused into the femoral veins of C57/BL6 mice, 5 min prior to tail biopsy; 0.5 cm of tail tip was excised, and blood collected in PBS, and time to cessation of bleeding was recorded. Graphs represent mean ± SEM, *n* = 10 per treatment; data were analysed by Student's *t*‐test, *P < 0.05

Thiol isomerase inhibition or ablation results in substantial disruption of thrombus formation in laser injury thrombosis models (Cho, Furie, Coughlin, & Furie, 2008; Holbrook et al., 2018; Holbrook et al., 2012; Passam et al., 2015; J. Zhou et al., 2017). The effects of an acute dose of zafirlukast (20 μM) on arterial thrombus formation were determined by intravital microscopy on cremaster muscle arterioles. Figure 4a shows time‐resolved representative images of thrombi formed in the presence of vehicle (0.1% [v/v] DMSO). Thrombi formed in the presence of zafirlukast (20 μM) are illustrated in Figure 4b. Figure 4c shows maximum fluorescence intensity measurements for vehicle (*n* = 18 thrombi, black circles) or zafirlukast‐treated mice (*n* = 12 thrombi, red squares); thrombus size was significantly impaired by zafirlukast treatment. Since significant and consistent effects were observed with 12 thrombi in the zafirlukast‐treated mice, no further mice were killed. Therefore this group had fewer replicates than the control but was still adequately powered. We assessed the effects of zafirlukast on bleeding time, measured by murine tail bleeding assay. Figure 4d shows that zafirlukast treatment (bleeding time of 243 s ± 31 s, *n* = 10, red squares) did not significantly prolong or shorten bleeding times compared to vehicle treatment (bleeding time of 225 s ± 28 s, *n* = 10, black circles).

### 3.4 Zafirlukast inhibits cell migration by mediation of cell‐surface thiols in MDA‐MB‐231 and HEK293T cells

In addition to the effects of thiol isomerases on the modulation of platelet integrin activation, thiol isomerases are known to be important for the modulation of integrin function in other cell types including vascular smooth muscle cells (Tanaka et al., 2019), lymphocytes (Bi, Hong, Lee, & Baum, 2011; Lawrence, Song, & Weber, 1996) and cancerous cells. Numerous studies have linked enhanced cell‐surface thiol isomerase expression with increased integrin‐dependent or mediated migration, invasion of surrounding tissue, and proliferation (Goplen et al., 2006; Hussmann et al., 2015; Li et al., 2016; Samanta et al., 2017; Xu et al., 2012; Xu et al., 2019). Cell migration and proliferation responses in scratch wound closure assays or transwell migration assays are known to be sensitive to thiol isomerase inhibition. The PDI inhibitors PACMA31, quercetin‐3‐rutinoside, bacitracin and anti‐PDI antibodies all impair wound closure through a mechanism that is proposed to include down‐regulation of thiol disulphide exchange in adhesion receptors containing β1 and β3 integrin subunits (Goplen et al., 2006; Popielarski, Ponamarczuk, Stasiak, Watala, & Swiatkowska, 2019). Similar effects have been observed in cells where PDI (Lin et al., 2016) or ERp57 expression has been silenced (Li et al., 2019; Ye, Fu, Dou, & Wang, 2018), suggesting that thiol isomerase expression is a pro‐migratory and pro‐survival factor and is targetable for disruption of cancer progression in a number of solid tumour and haematological cancer types.

We were keen to explore the effects of zafirlukast on integrin‐mediated cell migration in cell models where cells express CysLT receptor and are known to be sensitive to thiol isomerase inhibition (MDA‐MB‐231) and in cells lacking CysLT receptors (HEK293T). This would allow us to study cysteinyl LT (CysLT)‐independent actions of zafirlukast on redox‐sensitive integrin‐mediated function, which is proposed to be modulated by extracellular thiol isomerases.

Expression of the thiol isomerases PDI, ERp57, ERp72 and ERp5 was detected on “the surface of” non‐permeabilised (Figure S3) and permeabilised MDA‐MB‐231 cells (Figure S4). Using immunocytochemistry, staining for all enzymes was observed. Secondary antibody controls displayed no signal in the Alexa‐Fluor 488 channel. With permeabilization, some differences in staining were observed; PDI and ERp57 staining appeared to increase suggesting that although a small pool of cell‐surface thiol isomerase exists, a greater pool is retained within the cells.

**FIGURE 5:**
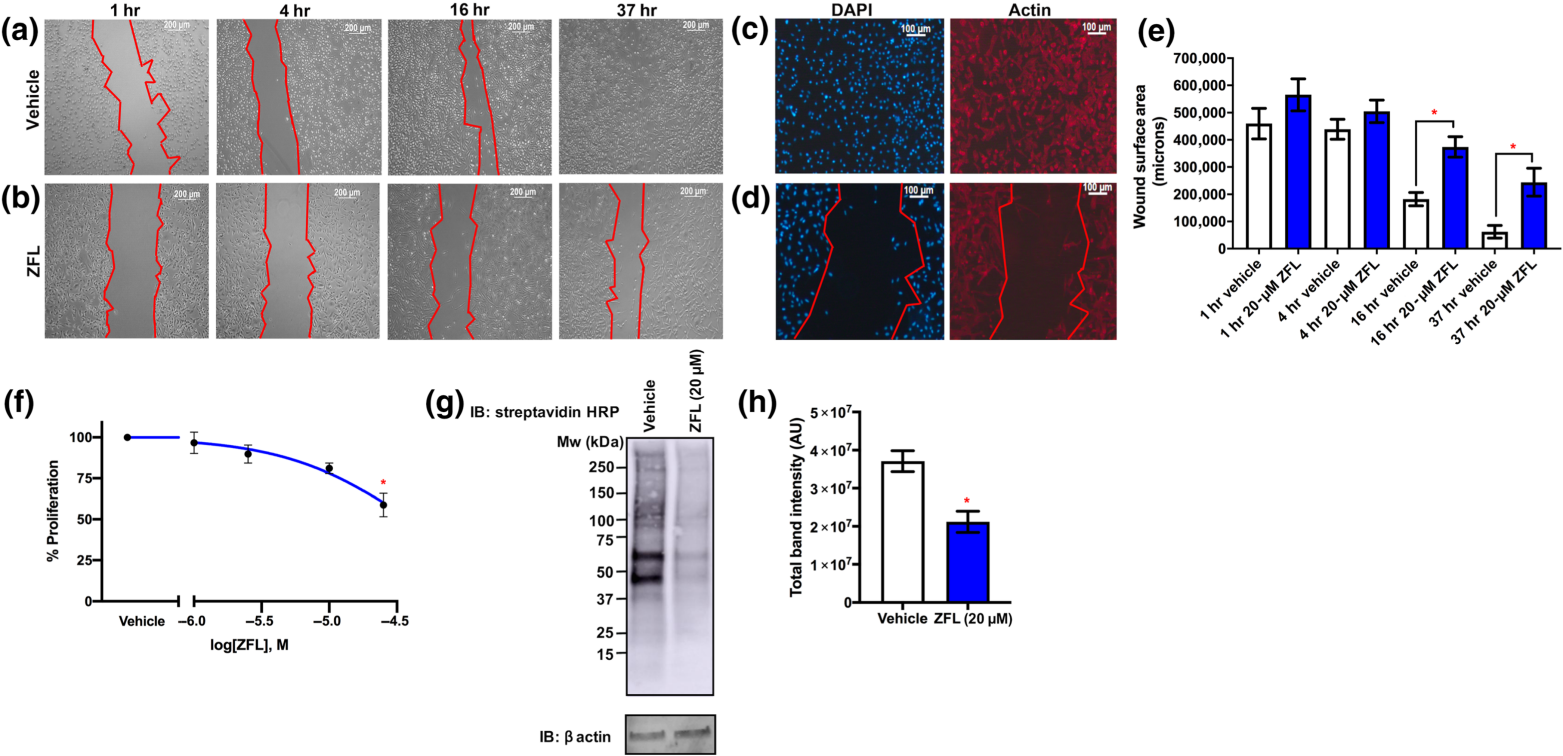
Thiol isomerase inhibition by zafirlukast (ZFL) prolongs scratch closure through modification of cell‐surface thiols in MDA‐MB‐231 cells. MDA‐MB‐231 seeded into wells of a 12‐well culture dish were grown to confluence. Wells were bisected by means of a vertical scratch, and detached cells removed by washing with PBS. (a) Vehicle or (b) zafirlukast (20 μM) was added to each well in the presence of DMEM supplemented with 1% (v/v) FBS, and images were recorded at 1, 4, 16, and 37 hr post‐scratch; cell leading edges are denoted in red. (c,d) At 37 hr post‐scratch, cells were fixed and permeabilised, then stained with phalloidin 568 for 1 hr, and following washes, counterstained with DAPI for 20 min. Cells were washed in PBS, and Vectashield H‐1000 was added prior to imaging. (e) Images were analysed using Zeiss Axiovision software, and closure areas calculated using Fiji ImageJ, *n* = 9. (f) Log proliferation data after 72‐hr treatment with zafirlukast of 1–30 μM (*n* = 6). (g) For thiol labelling, MDA‐MB‐231 seeded into wells of a 12‐well cell culture dish were grown to confluence, and vehicle or zafirlukast was added to each well in the presence of DMEM supplemented with 1% (v/v) FBS. Cells were washed 4 hr post‐treatment with PBS and free thiols labelled by incubation with MPB (100 μM) for 20 min. Free label was removed by multiple PBS washes and cells lysed in reducing sample treatment buffer for SDS‐PAGE analysis. Cell‐surface thiol labelling was detected using streptavidin‐HRP conjugate (1:10,000 dilution in 5% [w/v] BSA/TBS‐T), and chemiluminescence captured following the addition of ECL substrate. (h) Quantification of band intensities for surface thiol labelling (*n* = 9). Graphs represent mean ± SEM. Data were analysed by Student's *t*‐test, *P < 0.05

The effects of zafirlukast in integrin‐mediated cell migration were measured by scratch assay in which a scratch was inflicted on a monolayer of cells treated with either vehicle (Figure 5a) or zafirlukast (20 μM, Figure 5b), and images recorded until successful closure of vehicle‐treated scratch wells was achieved. Figure 5c,d shows nuclear and actin staining for wells at assay endpoint. Closure areas were measured at 1, 4, 16 and 37 hr (Figure 5e). Compared with area values obtained at 1 hr, vehicle‐treated cells had migrated to cover over 86% of the scratch area at endpoint (closure area of 62,163 ± 23,552) whereas with zafirlukast treatment, cell migration was significantly attenuated with 56% of the scratch area covered (closure area of 243,707 ± 51,057) at endpoint. The standard 3‐day growth inhibition assay was performed to determine if zafirlukast (1–30 μM) treatment was also able to impair proliferation. At the concentration used in the scratch assay (20 μM), zafirlukast had a minimal effect on cell proliferation and at a concentration of 30 μM, zafirlukast only modestly reduced proliferation by around 20% (Figure 5f).

In order to confirm that this effect was associated with decreased cell‐surface redox activity, labelling of surface free thiols was performed using the cell‐impermeant label MPB. At assay endpoint, cells were washed and labelled with MPB and following SDS‐PAGE separation, visualisation of the biotin moiety was performed using a streptavidin conjugate (Figure 5g). In vehicle‐treated samples, many bands were detected corresponding to multiple proteins containing free thiols. This was reduced by 42% in the zafirlukast‐treated lane consistent with changes in the redox state of cell‐surface proteins, specifically free thiols, implicating decreased thiol isomerase reductive function as a potential mode of action (Figure 5h).

MDA‐MB‐231 cells express CysLT_1_ and CysLT_2_ receptors, so in order to exclude or determine the role of CysLT_1/2_ receptor on the effects presented in Figure 5, similar experiments were performed in a cell line that does not possess these receptors. HEK293T are known to lack CysLT_1_ and LT_2_ receptor mRNA and as a consequence lack CysLT receptor signalling function (Atwood, Lopez, Wager‐Miller, Mackie, & Straiker, 2011; Figueroa, Kramer, Strange, & Denton, 2019). Similar to data presented for MDA‐MB‐231, expression of thiol isomerases was evident in both non‐permeabilised (Figure S5) and permeabilised cells (Figure S6). In permeabilised HEK293T, staining of PDI and ERp57 appear to be increased and change distribution compared to non‐permeabilised cells whereas ERp72 and ERp5 staining appear to decrease suggesting that a greater pool of these proteins is associated with the cell surface.

**FIGURE 6:**
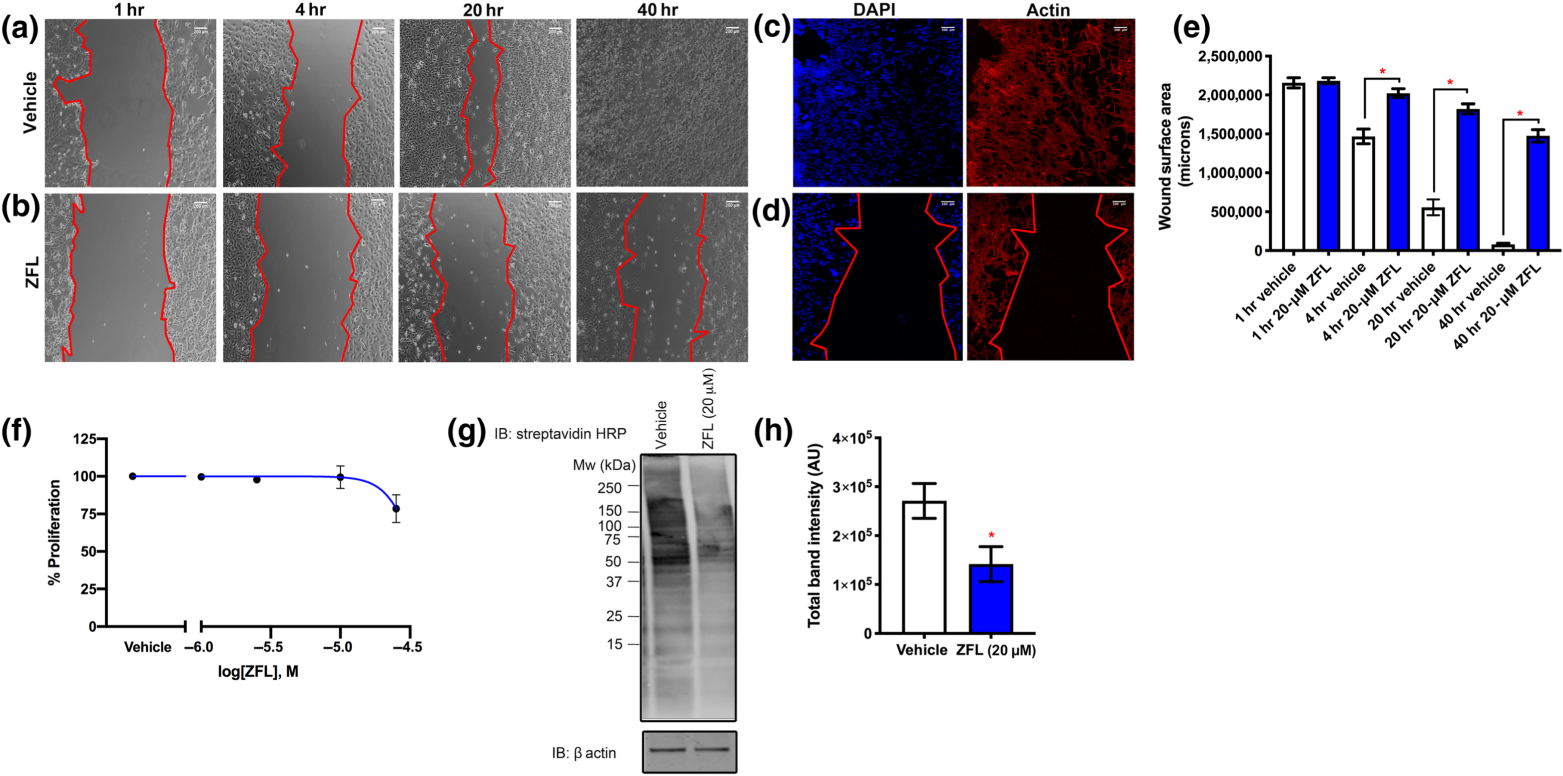
Zafirlukast (ZFL) impairs wound closure and thiol labelling in cells lacking CysLT receptors. HEK293T seeded into wells of a 12‐well culture dish (coated with fibronectin of 10 μg·ml^−1^) were grown to confluence. Wells were bisected by means of a vertical scratch, and detached cells removed by washing with PBS. (a) Vehicle or (b) ZFL (20 μM) was added to each well in the presence of DMEM supplemented with 1% (v/v) FBS, and images were recorded at 1, 4, 20 or 40 hr post‐scratch; cell leading edges are denoted in red. (c,d) At 40 hr post‐scratch, cells were fixed and permeabilised, then stained with phalloidin 568 for 1 hr, and following washes, counterstained with DAPI for 20 min. Cells were washed in PBS, and Vectashield H‐1000 was added prior to imaging. (e) Images were analysed using Zeiss Axiovision software, and closure areas calculated using Fiji ImageJ, *n* = 12. (f) Log proliferation data after 72‐hr treatment with zafirlukast of 1–30 μM (*n* = 6). (g) For surface thiol labelling, HEK293T seeded into wells of a 12‐well cell culture dish were grown to confluence, and vehicle or zafirlukast was added to each well in the presence of DMEM supplemented with 1% (v/v) FBS. Cells were washed 4 hr post‐treatment with PBS and free thiols labelled by incubation with MPB (100 μM) for 20 min. Free label was removed by multiple PBS washes and cells lysed in reducing sample treatment buffer for SDS‐PAGE analysis. Cell‐surface thiol labelling was detected using streptavidin‐HRP conjugate (1:10,000 dilution in 5% [w/v] BSA/TBS‐T), and chemiluminescence captured following the addition of ECL substrate. (h) Quantification of band intensities for surface thiol labelling (*n* = 7). Graphs represent mean ± SEM. Data were analysed by Student's *t*‐test, *P < 0.05

In order to explore the effects of zafirlukast on thiol isomerase‐mediated cell migration in HEK293T, scratch assays were performed, and closure areas recorded at 1, 4, 20 and 40 hr for (Figure 6a) vehicle and (Figure 6b) zafirlukast. At 40 hr post‐scratch, vehicle‐treated cells had over 96% coverage (closure area of 80,686 [±15,705]), whereas zafirlukast treatment attenuated migration to 32% (1,475,154 [±81,061] of the initial area, Figure 6e). Impairment of HEK293T cell proliferation was not detected after treatment of cells with (1–30 μM) of zafirlukast (Figure 6f). Surface MPB labelling was also decreased in HEK293T treated with zafirlukast when compared to vehicle levels (Figure 6g); zafirlukast caused a 47% decrease in labelling compared with vehicle treatment (Figure 6h). These data highlight the importance of thiol isomerases in cell migration and demonstrate that zafirlukast is an effective inhibitor of thiol isomerase‐mediated effects in both the presence and absence of functional CysLT_1_/LT_2_ signalling pathways.

## 4 DISCUSSION

The pro‐inflammatory Cysteinyl leukotrienes (CysLT) (CysLTC4, LTD4 and LTE4) are formed through the 5‐lipoxygenase (5‐LOX) arachidonic acid metabolism pathway as a component of host defence in immune cells. Immune and inflammatory diseases such as asthma are often associated with overproduction of CysLT which bind to CysLT receptors and induce immune‐type signalling. Pharmacological antagonism of CysLT receptors (CysLT_1_ and CysLT_2_) by drugs such as zafirlukast and montelukast is a common approach for the treatment of chronic asthma.

Zafirlukast was first used in 1996 for the antagonism of CysLT_1_ receptors present on the human airway smooth muscle cells to prevent the pro‐inflammatory effects of CysLT binding. Zafirlukast is generally regarded to be well tolerated in both animal safety studies and in humans with only minimal side effects being reported for the majority of patients. Some studies have demonstrated that hepatic clearance of zafirlukast from the plasma is reduced in patients with cirrhosis of the liver and in elderly patients, so treatment of these patients with zafirlukast is not advised. Zafirlukast is metabolised exclusively in the liver by the cytochrome P450 isoenzyme CYP2C9. Liver clearance of zafirlukast can be altered by co‐administration with the drugs erythromycin, theophylline or terfenadine, which reduce plasma zafirlukast levels while aspirin enhances its plasma levels, Therefore these drugs are not recommended in patients receiving zafirlukast treatment (Dekhuijzen & Koopmans, 2002).

Clinical efficacy is observed when oral doses of 20 mg are given twice daily and higher doses are tolerated. This dose has been shown to result in a peak plasma concentration (cMax) of 697 ng·ml^−1^ at 3 hr, which is equivalent to a plasma concentration of 1.2 μM (Fischer et al., 2000).

Platelets do not produce CysLT as they lack 5‐LOX and therefore are unable to convert arachidonic acid to CysLTs unless they are interacting with immune cells that can generate the lipid precursor (Laidlaw & Boyce, 2012). Healthy human donor platelets have been shown to express the CysLT receptors CysLT_1_ and CysLT_2_, a small pool of which are found on the cell surface (Hasegawa et al., 2010). Following stimulation with purified CysLT, release of the eosinophil chemotactic factor RANTES (CCL5) from platelet α‐granule stores can be detected suggesting that under inflammatory conditions, where platelets are circulating in proximity to cells producing CysLT, CysLT receptor stimulation can drive the secretion of platelet stored inflammatory mediators (Hasegawa et al., 2010). However the function of CysLT receptors or the effects of zafirlukast on platelet function under basal, non‐inflammatory conditions is not known and is likely only to be determined if CysLT_1_ null mouse platelets were available.

Through compound library screening for small molecule inhibitors of thiol isomerases, we identified zafirlukast and montelukast as candidate inhibitors of multiple thiol isomerase family members in addition to their anti‐asthma function. Conventionally, the activity and functional role of thiol isomerases has been established using function‐blocking antibodies or knockout mouse models. These studies have not only revealed the importance of thiol isomerase activity in the regulation of thrombosis and haemostasis but also provided insight into the subtle differences in platelet signalling targeted by each thiol isomerase enzyme. In recent years, the focus has shifted onto the development of pharmacological thiol isomerase inhibitors with characterisation of several inhibitors of protein disulphide isomerase forthcoming, some of which have entered into clinical trials. No broad‐spectrum thiol isomerase inhibitors with similar potency for multiple enzymes that are potent anti‐thrombotics and have proven tolerance in vivo have been developed. Our study identifies zafirlukast and montelukast to be novel inhibitors of thiol isomerase activity, and we tested the functional consequences of pan‐thiol isomerase inhibition in human and murine platelets.

Zafirlukast treatment diminished platelet aggregatory responses and granule secretion in a concentration‐dependant manner. In addition to the effects on granule secretion, zafirlukast impaired calcium mobilisation and fibrinogen ligation to integrin α_IIb_β_3_. Using a murine thrombosis model, we were able to demonstrate that zafirlukast diminished thrombosis in vivo but did not impair or enhance bleeding suggesting that pan‐thiol isomerase inhibition is tolerated and is a viable therapeutic option, worthy of further exploration as many currently prescribed anti‐thrombotic therapies inhibit platelet function but also cause bleeding.

Differential regulation effects of inhibition of multiple thiol isomerases between different functional assays were evident with dose response profiles not being identical in the effects observed. IC_50_ values obtained for platelet aggregation were around fourfold lower (1.66 μM) than required to elicit the same level of inhibition of dense granule secretion. Similarly, α‐granule secretion required concentrations of 5‐μM zafirlukast or more to cause significant inhibition. Previous studies have highlighted differential effects of this nature; antibody‐mediated inhibition of ERp5 or ERp72 results in dysfunctional α‐granule secretion (Holbrook et al., 2018; Jordan et al., 2005), whereas little or no effect is observed with PDI or ERp57 blockade (Holbrook et al., 2012; Kim et al., 2013). Likewise, calcium flux is regulated by ERp57 and ERp72 but not PDI or ERp5. In in vivo assays, all thiol isomerases have been shown to be critical for initial thrombus formation and propagation; however, curiously not all thiol isomerases are critical for haemostasis in mice. ERp57 and ERp72 ablation results in prolonged tail bleeding times (Wang et al., 2013; J. Zhou et al., 2017), while, as for zafirlukast treatment of mice, PDI‐deficient mice do not show a haemostasis defect (Kim et al., 2013). We propose that this may be a consequence of different effects of each enzyme on individual aspects of platelet activation balanced with different enzymes being inhibited at different zafirlukast concentrations. Therefore, non‐overlapping aspects of platelet regulation may require greater levels of inhibitor to counterbalance the sensitive to inhibition and insensitive to inhibition effects of each enzyme in the multiple pathways of platelet activation.

Thiol isomerases are also important for mediating integrin conformational changes associated with cell adhesion and migration. We therefore explored the effects of zafirlukast on these processes using a breast cancer cell line MDA‐MB‐231, which has been previously shown to be sensitive to thiol isomerase inhibition. Since HEK293T cells do not express CysLT receptors, experiments were repeated in these cells to determine if responses are dependent on the CysLT_1_ receptor. We demonstrate that zafirlukast is able to impair cell migration regardless of CysLT receptor expression, without impairing proliferation, and that zafirlukast treatment results in a decrease in cell‐surface thiol levels consistent with zafirlukast eliciting redox changes at the cell surface. Immunoblotting for surface thiol‐biotinylation revealed labelling changes in many proteins which may be substrates or targets of thiol isomerases, proteins of great future interest to identify.

Whether zafirlukast acts on other protein targets other than thiol isomerases in platelets, HEK293T or MDA‐MB‐231 cells remain to be determined. In bronchial epithelial and astrocytoma cell lines, CysLT_1_ antagonists can reduce PKA signalling following nucleotide stimulation of the P2Y_1_ purinergic receptor pathway. It is not clear if this was through direct binding to the receptor or as a consequence of indirect effects on cell signalling (Lau, Chow, Au, & Ko, 2011; Mamedova et al., 2005). Similarly, recent findings suggest that zafirlukast can directly activate PPARγ signalling in adipocytes (Gobel et al., 2019). In platelets, PPARγ ligands negatively regulate platelet responses (Moraes et al., 2010). The potential effects of zafirlukast on these pathways in platelets remain to be explored and cannot be ruled out as additional mechanisms of zafirlukast action on platelet function. Since the onset of this study, recent publications report that zafirlukast may also be beneficial for the treatment of endothelial dysfunction, inflammation (X. Zhou, Cai, Liu, Wu, & Gao, 2019), glioblastoma (Piromkraipak et al., 2018) and bladder cancer (Nguyen et al., 2018). It would be interesting to establish if these effects are related to the inhibition of thiol isomerase activity.

Most inhibitors of thiol isomerases characterised to date act by competitive binding to the substrate binding domains of the enzyme; therefore, future studies may entail a detailed kinetic analysis of each enzyme to determine how zafirlukast binds and modifies its activity. Since zafirlukast is able to inhibit at least five enzymes from a family of at least 20 thiol isomerase proteins, such elaborate studies were beyond the scope of this study.

In summary, we provide evidence that the anti‐asthma compound zafirlukast is a potent pan‐thiol isomerase inhibitor which modulates platelet function, cell migration, and thrombosis but does not show detrimental effects on haemostasis.

## AUTHOR CONTRIBUTIONS

L.H., S.K., P.S., S.N., J.G., and E.B. performed experiments. L.H. and S.R. performed analysis. Library screening assays were performed by D.K., M.P., and C.V. L.H. prepared the manuscript. L.H., S.K., D.K., and J.M.G. designed experiments, and L.H., D.K., and J.M.G. proofed the manuscript.

## CONFLICT OF INTEREST

The authors declare no conflicts of interest.

## DECLARATION OF TRANSPARENCY AND SCIENTIFIC RIGOUR

This Declaration acknowledges that this paper adheres to the principles for transparent reporting and scientific rigour of preclinical research as stated in the *BJP* guidelines for Natural Product Research, Design & Analysis, Immunoblotting and Immunochemistry and Animal Experimentation, and as recommended by funding agencies, publishers, and other organisations engaged with supporting research.

## Supporting information


**Figure S1.** Montelukast also shows broad spectrum thiol isomerase (TI) inhibitory characteristics

**Figure S2.** Zafirlukast inhibits platelet aggregation

**Figure S3.** Immunofluorescence analysis of thiol isomerase (TI) expression in non‐permeabilised MDA‐MB‐231

**Figure S4.** Immunofluorescence analysis of thiol isomerase (TI) expression in permeabilised MDA‐MB‐231

**Figure S5.** Immunofluorescence analysis of thiol isomerase (TI) expression in non‐permeabilised HEK293T

**Figure S6.** Immunofluorescence analysis of thiol isomerase (TI) expression in permeabilised HEK293T

## Data Availability

The data that support the findings of this study are available from the corresponding author upon reasonable request. Some data may not be made available because of privacy or ethical restrictions.
