# Supplementary figures and images for: Zafirlukast is a broad‐spectrum thiol isomerase inhibitor that inhibits thrombosis without altering bleeding times
Source: Br J Pharmacol. 2021 Jan 4;178(3):550–63. doi: 10.1111/bph.15291 (PMC9328650; doi:10.1111/bph.15291)

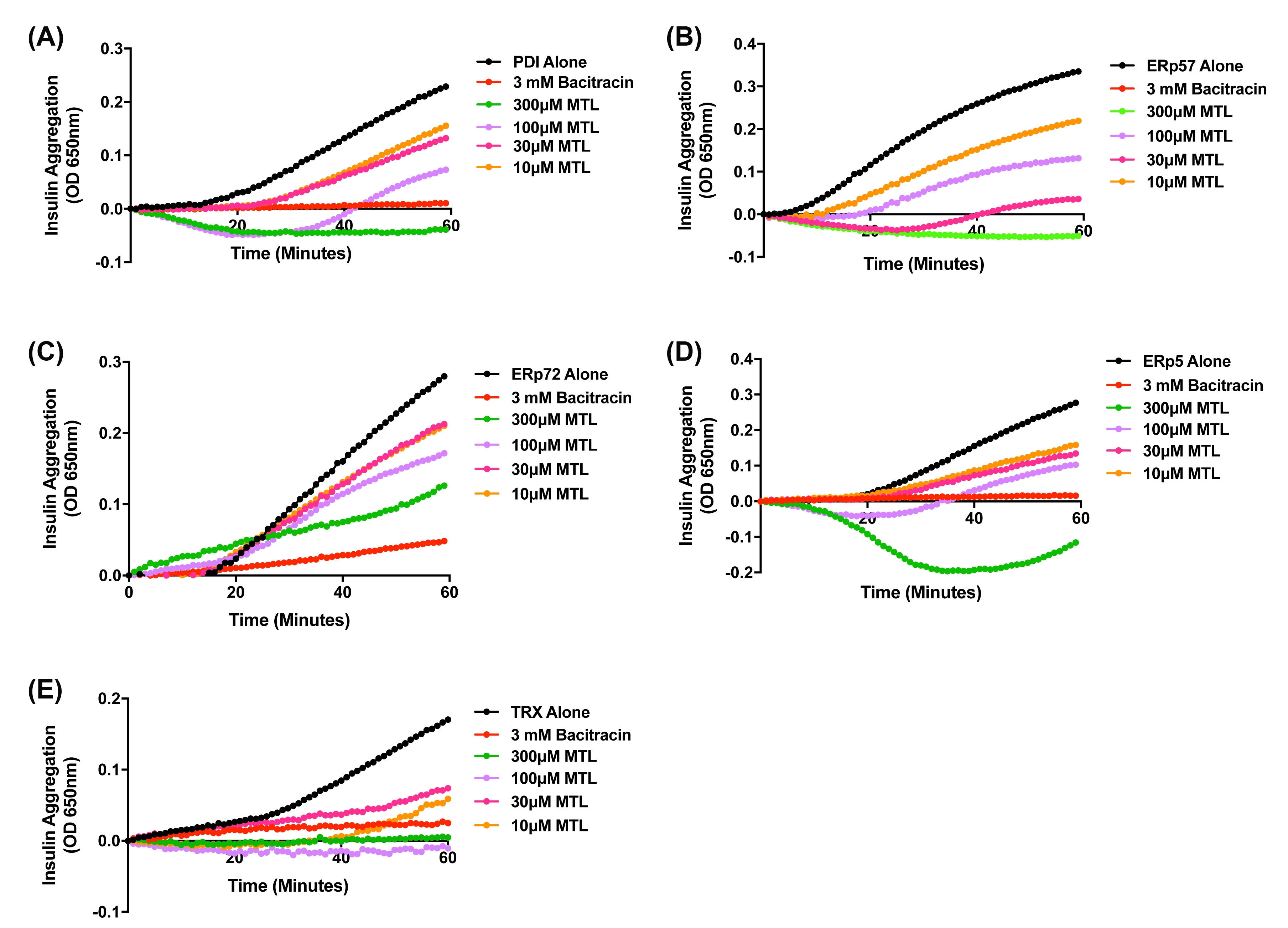

Supplement: Supplementary file 1 — Figure S1. Montelukast also shows broad spectrum thiol isomerase (TI) inhibitory characteristics [file BPH-178-550-s005.tif]

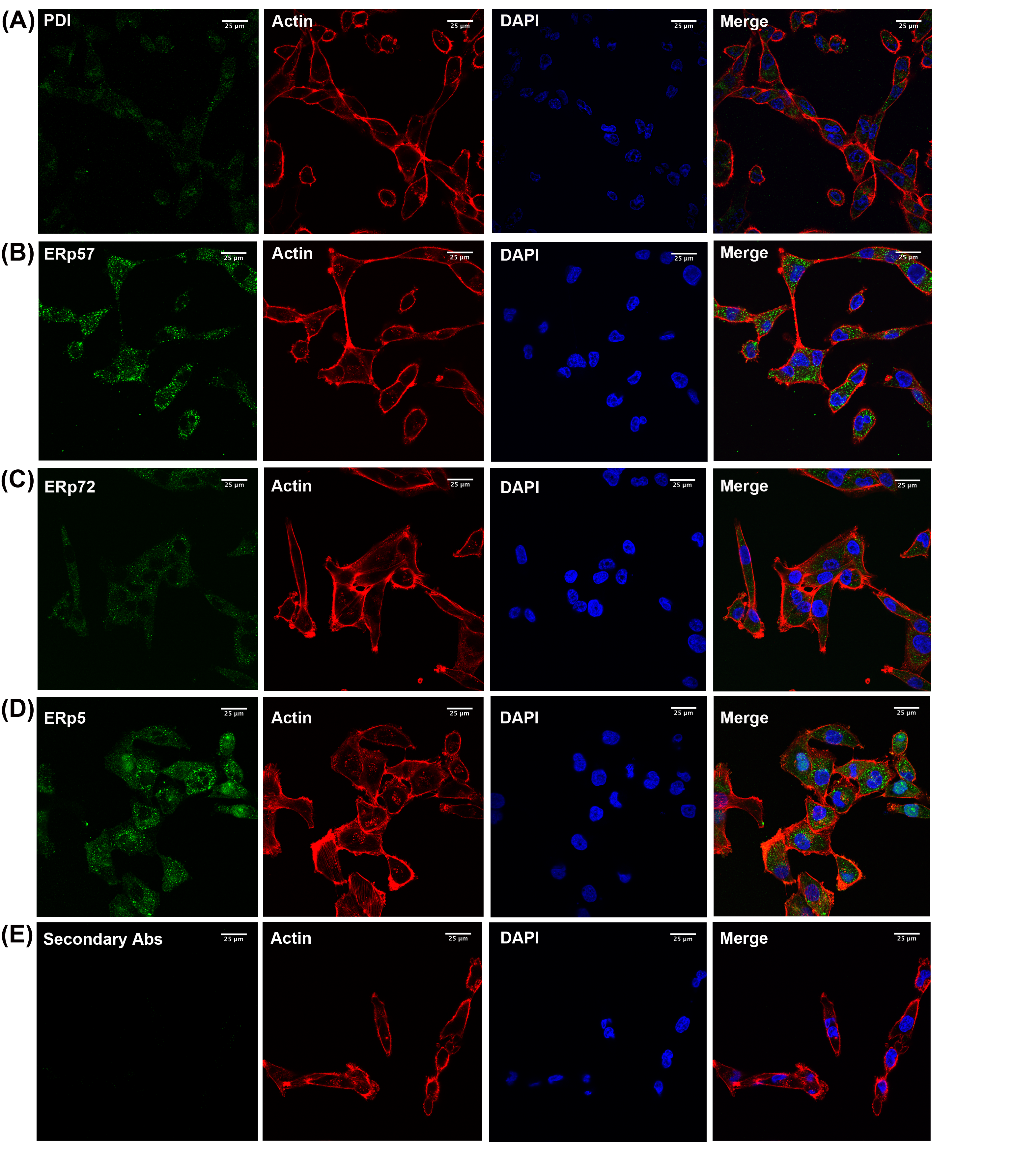

Supplement: Supplementary file 2 — Figure S2. Zafirlukast inhibits platelet aggregation [file BPH-178-550-s004.tif]

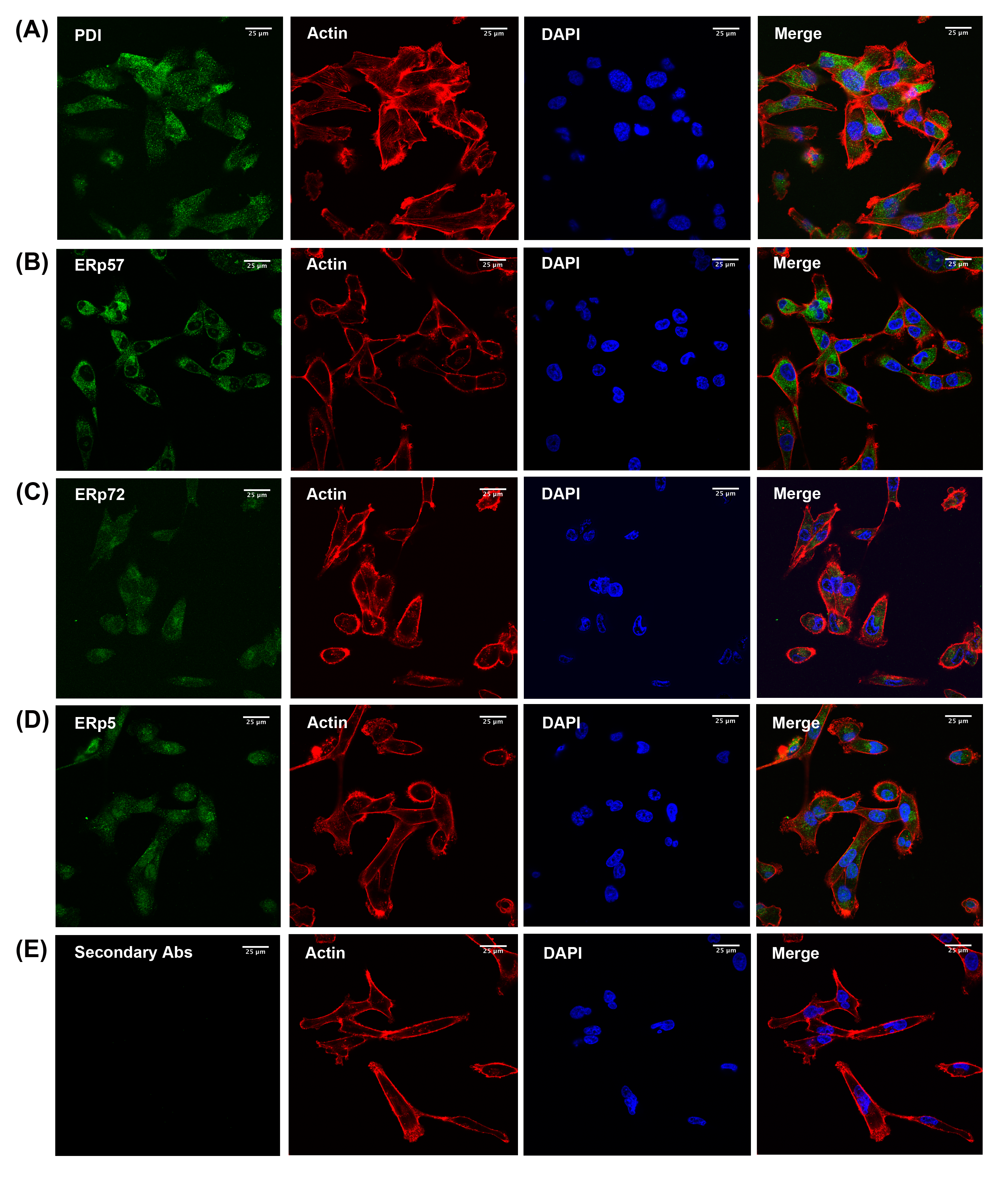

Supplement: Supplementary file 3 — Figure S3. Immunofluorescence analysis of thiol isomerase (TI) expression in non‐permeabilised MDA‐MB‐231 [file BPH-178-550-s003.tif]

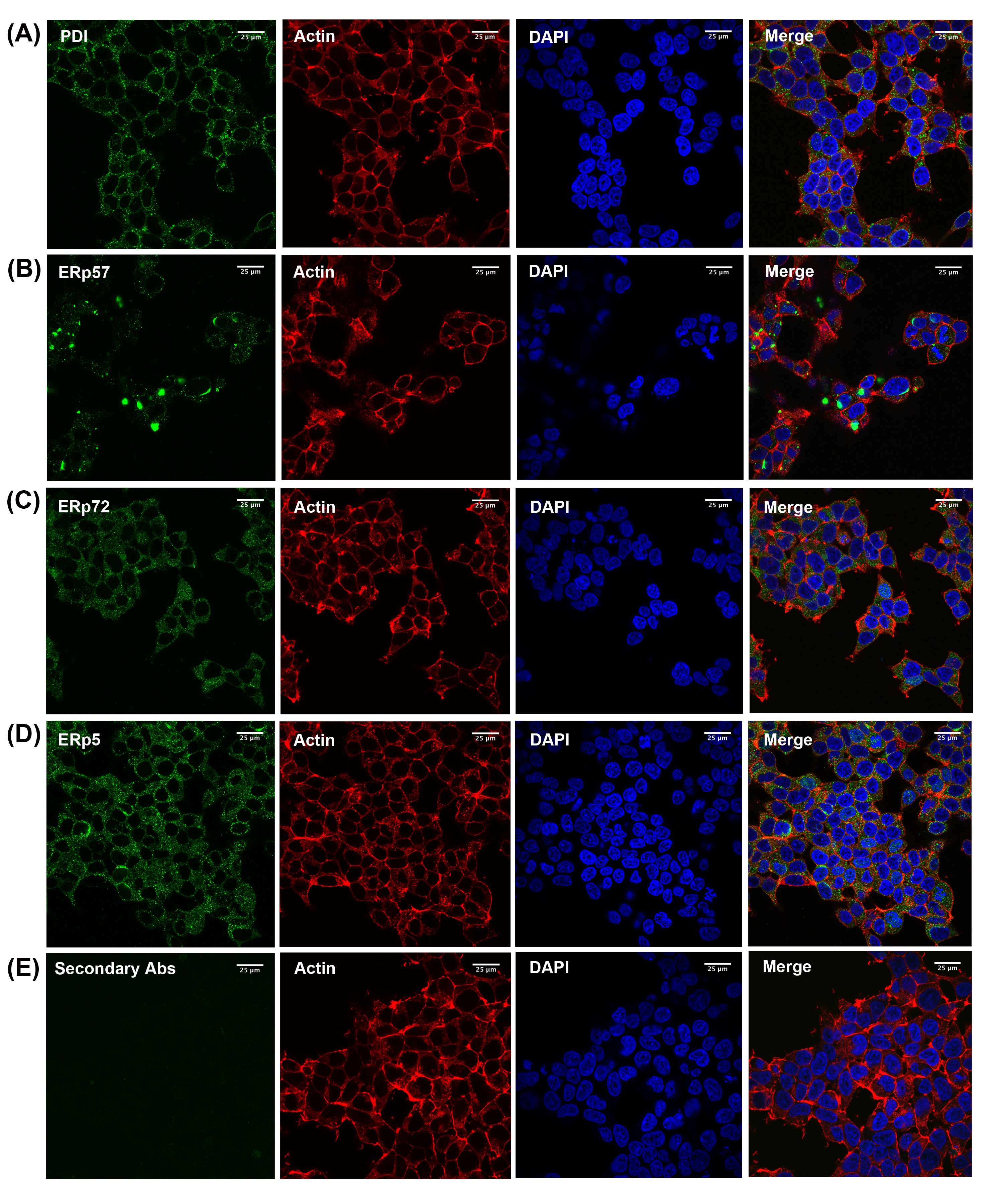

Supplement: Supplementary file 4 — Figure S4. Immunofluorescence analysis of thiol isomerase (TI) expression in permeabilised MDA‐MB‐231 [file BPH-178-550-s002.tif]

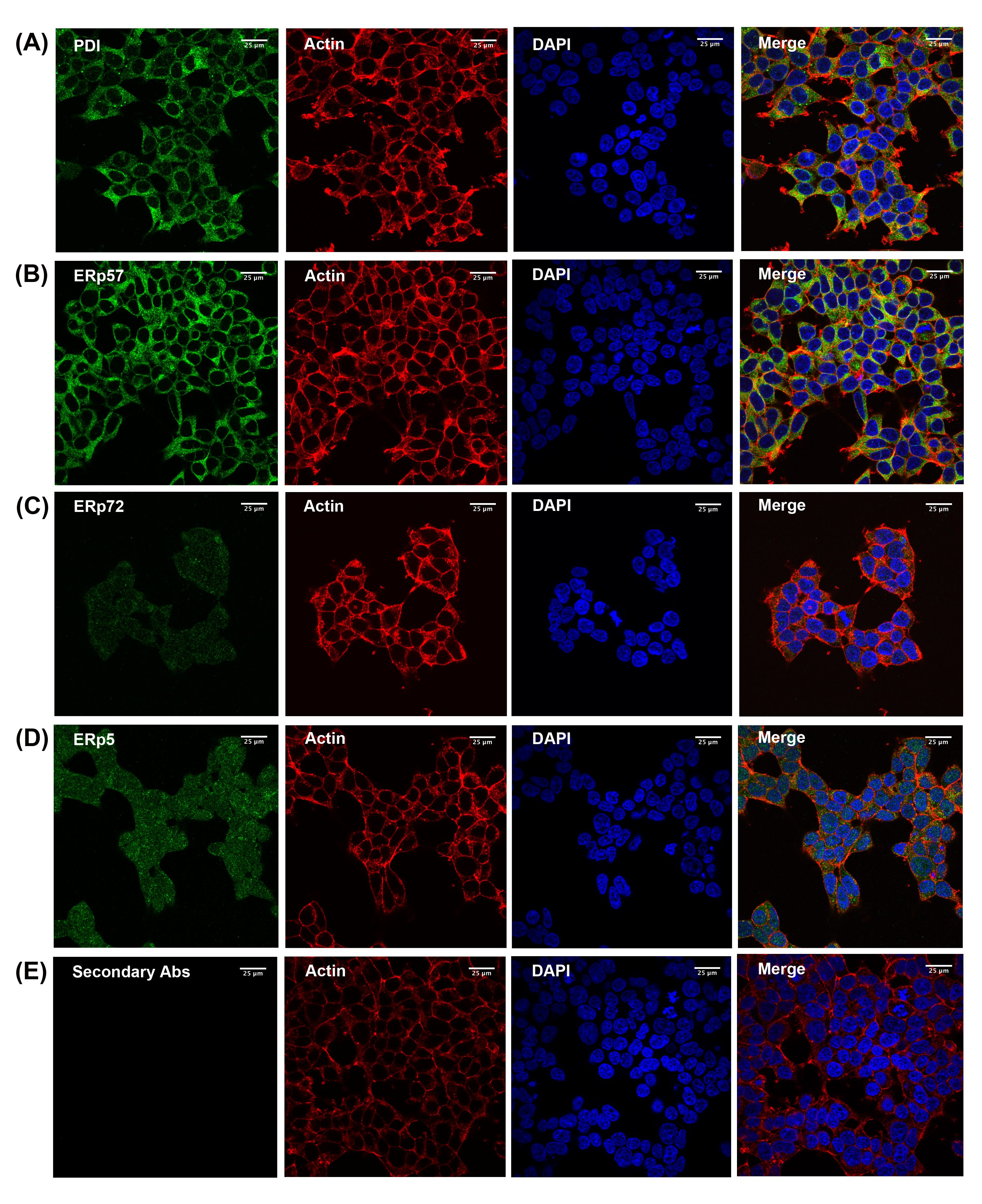

Supplement: Supplementary file 5 — Figure S5. Immunofluorescence analysis of thiol isomerase (TI) expression in non‐permeabilised HEK293T [file BPH-178-550-s001.tif]

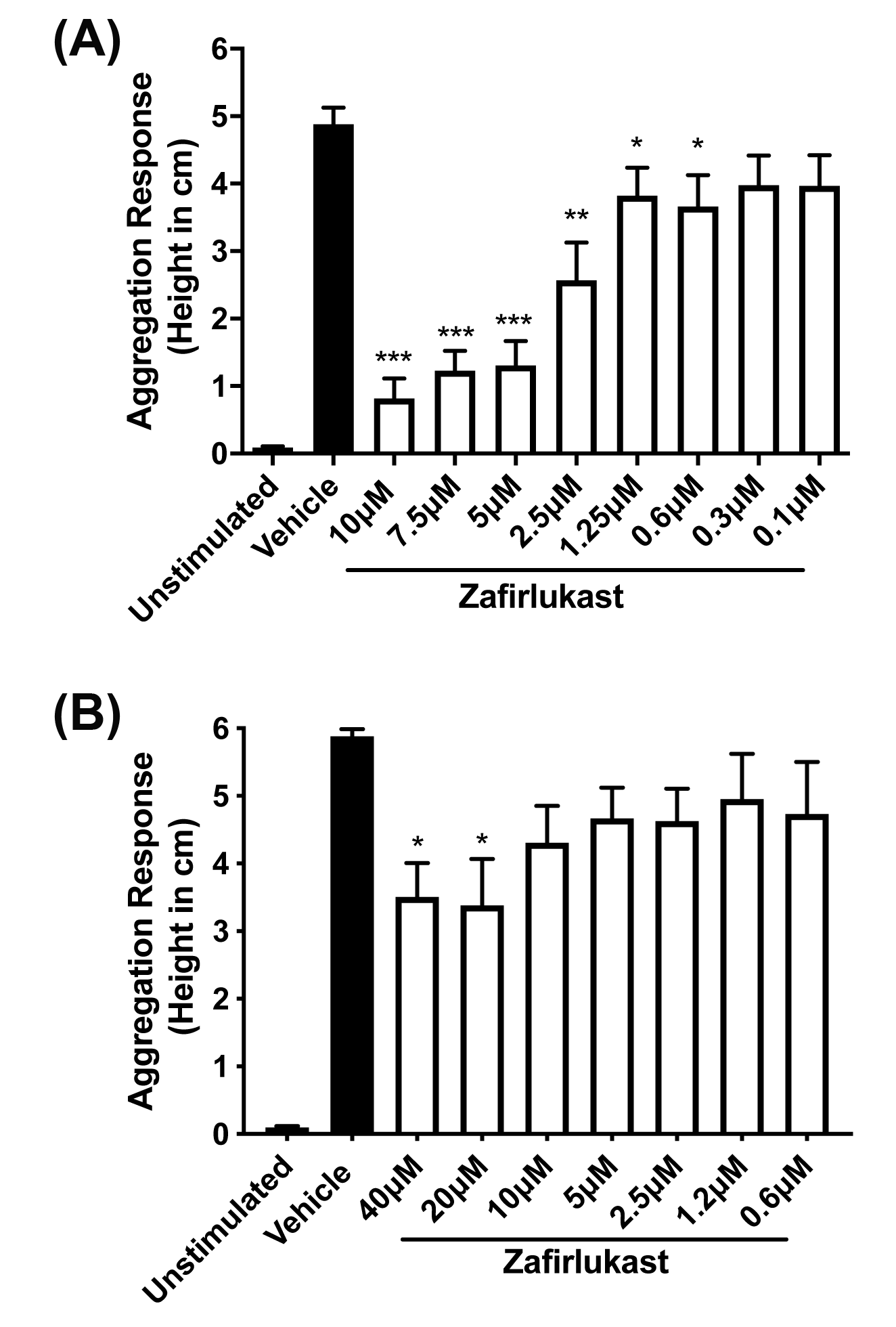

Supplement: Supplementary file 6 — Figure S6. Immunofluorescence analysis of thiol isomerase (TI) expression in permeabilised HEK293T [file BPH-178-550-s006.tif]
